# Reactive oxygen species and sperm DNA damage in infertile men presenting with low level leukocytospermia

**DOI:** 10.1186/1477-7827-12-126

**Published:** 2014-12-19

**Authors:** Ashok Agarwal, Aditi Mulgund, Saad Alshahrani, Mourad Assidi, Adel M Abuzenadah, Rakesh Sharma, Edmund Sabanegh

**Affiliations:** Center for Reproductive Medicine, Cleveland Clinic, Cleveland, OH 44195 USA; Salman Bin Abdul Aziz University, College of Medicine, Al Kharj, Jeddah, Saudi Arabia; Center of Excellence in Genomic Medicine Research, King AbdulAziz University, Jeddah, Saudi Arabia; KACST Technology Innovation Center in Personalized Medicine, King AbdulAziz University, Jeddah, Saudi Arabia

**Keywords:** Male infertility, Low leukocytospermia, Oxidative stress, Reactive oxygen species, DNA fragmentation

## Abstract

**Background:**

Leukocytes contribute directly and indirectly to reactive oxygen species (ROS) production. Although leukocytospermia is defined as the presence of ≥1 × 10^6^ white blood cells/mL (WBC/mL) in a semen sample, the presence of less than 1×10^6^ WBC/mL (low-level leukocytospermia) can still produce a detectable amount of ROS, impairing sperm function and lowering the chances of pregnancy. Our objective was to assess the effect of low-level leukocytospermia on semen quality, ROS levels, and DNA damage in infertile men.

**Methods:**

Semen samples were examined from 472 patients and divided into 3 groups: no seminal leukocytes; group 2, men with low-level leukoctyospermia (0.1-1.0 × 10^6^ WBC/mL); and group 3, frank leukocytospermia, (>1.0 × 10^6^. WBC/mL). Semen analysis, leukoctyospermia, reactive oxygen species and DNA fragmentation was tested.

**Results:**

Conventional semen parameters between the 3 groups were similar. Group 2 patients had significantly higher levels of ROS and sperm DNA fragmentation (1839.65 ± 2173.57RLU/s; DNA damage: 26.47 ± 19.64%) compared with group 1 (ROS: 1101.09 ± 5557.54 RLU/s; DNA damage: 19.89 ± 17.31%) (ROS: p = 0.002; DNA damage: p = 0.047). There was no significant difference in ROS levels between groups 2 and 3.

**Conclusions:**

Patients presenting with low-level leukocytospermia have seminal oxidative stress. Although these patients are not categorized as leukocytospermic by current World Health Organization (WHO) guidelines, these men may benefit by treatment with antibiotics, testing for bacterial cultures, or antioxidant supplements to reduce ROS-induced sperm DNA fragmentation and improve their chances of fertility. The WHO guidelines for leukocytospermia may need to be revised accordingly.

## Background

Leukocytospermia refers to the presence of leukocytes in semen. It is defined by the World Health Organization (WHO) as ≥1 × 10^6^ WBC/mL of semen [1] and is present in 10% to 20% of infertile men [2]. The condition can be an indicator of male genital tract infection or inflammation. However, in the absence of infection, it may originate from etiologies such as cigarette smoking, heavy alcohol use, or age [3–6].

Leukocytes (polymorphonuclear neutrophils and macrophages) have important implications in male fertility in that they produce reactive oxygen species (ROS). At low levels, ROS play a physiologic role [7], but at higher levels, they cause oxidative stress, which overwhelms the physiological mechanisms of sperm and causes damage. This damage has been established to occur via lipid peroxidation of the plasma membrane [8]. After gaining entry into the sperm, ROS target genetic materials, destroying mitochondrial DNA and inhibiting intracellular ATP production [9, 10]. Without proper ATP production, both functionality and sperm motility is affected [11]. As a result, male infertility can occur. Oxidative stress can also decrease success rates of assisted reproduction procedures such as in vitro fertilization (IVF) and intracytoplasmic sperm injection (ICSI) [12, 13].

The WHO threshold for leukocytospermia has previously been challenged as being too high due to the detrimental effects seminal leukocytes have on sperm at lower levels [14–17]. Leukocyte counts lower than 1.0 × 10^6^ WBC/mL (low-level leukocytospermia) has been shown to cause a significant decrease of motility and DNA integrity [15]. In addition, previous studies have reported high bacterial counts at low levels of leukocytospermia [14], abnormal sperm morphology at levels of leukocytospermia as low as 0.5 × 10^6^ WBC/mL [18], and impaired sperm motility with significantly increased levels of cytokines IL-6 and IL-8 in semen with moderately increased leukocytes [19].

Therefore, our objective was to assess the effects of low-level leukocytospermia on semen quality and oxidative stress markers (ROS levels and DNA damage) in infertile men in an effort to examine whether the WHO definition of leukocytospermia should be lowered.

## Methods

### Subjects

This study was approved by Cleveland Clinic review board, and all patients provided informed consent. This observational study used semen samples collected in accordance with a prospective protocol. Between January 2012 and April 2013, 472 infertile patients presenting to the Cleveland Clinic Center for Male Fertility underwent assessment, which included semen analysis. Patients were excluded if they had azoospermia. The following clinical data were recorded: age, body mass index (BMI), volume of semen ejaculate, period of sexual abstinence, duration of infertility, primary or secondary infertility, and history of smoking and marijuana and alcohol use.

Semen samples were examined for sperm concentration, motility and morphology, seminal leukocyte levels via peroxidase or the Endtz test, reactive oxygen species by chemiluminescence assay, and sperm DNA damage by TUNEL assay. The patients were divided into three groups based on their seminal leukocyte levels: group 1, no seminal leukocytes; group 2, men with low-level leukocytospermia, defined as 0.1-1.0 × 10^6^ WBC/mL; and group 3, frank leukocytospermia, defined by the WHO as >1.0 × 10^6^ WBC/mL.

### Semen analysis

Computer-assisted semen analysis was performed in our andrology laboratory using a Hamilton-Thorne-Integrated Visual Optical System, version 10, semen analyzer (Hamilton Thorne Biosciences, Beverly, MA). A 5-μL aliquot was loaded onto a counting chamber for each semen parameter analyzed, and 4–10 fields were examined manually and by computer-assisted semen analysis. Sperm concentration, motility and morphology using the strict criteria of Kruger et al. were reported according to 2010 WHO criteria [1].

### Seminal leukocyte quantification

To distinguish the WBCs from immature sperm, only samples with >5 round cells in high power field (hpf) were assessed with peroxidase staining (Endtz test) [20]. Peroxidase-positive leukocytes staining brown were counted using a Makler’s counting chamber (Sefi Medical, Haifa, Israel) under bright-field microscopy and results reported as ×10^6^ WBC/mL semen.

### Reactive oxygen species

Production of ROS was measured by the chemiluminescence assay method using luminol (5-amino-2,3-dihydro- 1,4-phthalazinedione; Sigma Chemical, St. Louis, MO) as the probe. Ten microliters of 5 mmol/L luminol prepared in dimethyl sulfoxide (Sigma Chemical) were added to 400 μL of the washed sperm suspension. Levels of ROS were determined by measuring chemiluminescence with an Autolumat LB 953 luminometer (AutoLumat Plus LB 953, Oakridge, TN) in the integrated mode for 15 minutes. The results were expressed as Relative Light Units/sec/ 10^6^ sperm (hereafter referred as RLU/s) [21].

### Measurement of sperm DNA damage

Sperm DNA damage was quantified using the terminal deoxynucleotidyl transferase (TdT)-mediated fluorescein-dUTP nick end labeling (TUNEL) assay kit (Apo-Direct; Pharmingen, San Diego, CA) [22]. This is based on the principal that TdT binds to the 3′-hydroxyl (OH) termini of the single and double strand DNA breaks. It is labeled with, fluorescein isothiocyanate tagged 2′-deoxyuridine, 5′-triphosphate nucleotides (FITC-dUTP) followed by flow cytometry. Briefly, after fixation with 3.7% paraformaldehyde for 30 minutes on ice, spermatozoa were washed and resuspended in ice cold 70% ethanol and then in phosphate buffered saline. The specimens were centrifuged at 1600 rpm for 7 minutes, and the pellet was resuspended for 60 minutes at 37°C in 50 μL of a staining solution containing terminal deoxynucleotidyl transferase (TdT) enzyme, TdT reaction buffer, FITC-dUTP and distilled water. Both negative (without TdT) and positive samples (DNase I) were included. After centrifugation, the cells were washed twice in rinse buffer, resuspended in 0.5 mL of propidium iodide (PI)/ RNase solution, and incubated for 30 minutes in the dark at room temperature in anticipation of flow cytometry [23]. PI stains total DNA and FITC-dUTP stains apoptotic cells.

### Flow cytometry analysis

All fluorescence signals of labeled spermatozoa were analyzed by the flow cytometer FACScan (Becton Dickinson). A minimum of 10,000 spermatozoa were examined for each assay at a flow rate of <100 cells/second. The sperm population was gated using 90° and forward-angle light scatter to exclude debris and aggregates. The excitation wavelength was 488 nm supplied by an argon laser at 15 mW. PI red fluorescence (580–630 nm) was analyzed in the FL-2 channel. The percentage of PI-positive cells and the mean fluorescence were calculated on a 1023-channel scale and analyzed using the flow cytometer software FlowJo, version 6.4.2 (FlowJo, LLC, Ashland, OR) [23].

### Statistical analysis

Group comparisons were performed with respect to categorical variables using chi-square or Fisher’s exact tests. Comparisons of greater than 2 groups with respect to quantitative variables were performed with Kruskal-Wallis tests, while 2-group comparisons were performed using the Wilcoxon Rank Sum test [24]. *P* < 0.05 was considered statistically significant.

## Results

**Figure 1:**
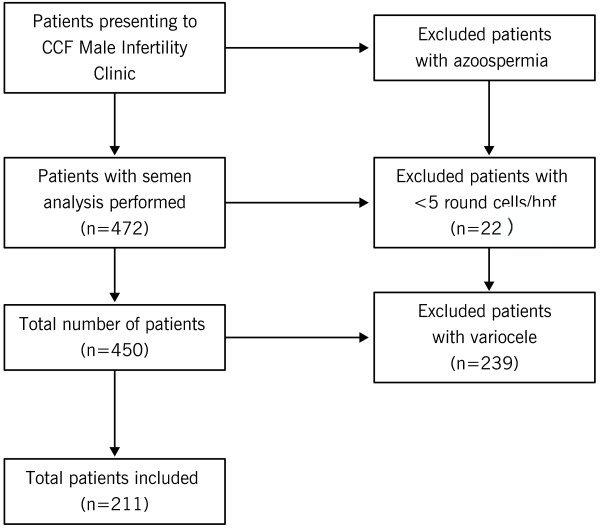
**Flow chart demonstrating inclusion and exclusion criteria.** This chart demonstrates that while 472 patients initially presented with infertility, only 211 were included in this study. 22 were excluded due to too few round cells present in their semen, and 239 patients were excluded due to their history of varicocele. Patients included were of reproductive age, and had been deemed infertile between 1–12 years.

Between January 2012 and April 2013, 472 infertile patients presented to the Cleveland Clinic Center for Male Fertility; inclusion and exclusion criteria are shown in Figure 1. Two hundred and eleven eligible patients were examined. Endtz testing categorized these patients as follows: group 1, no leukocytospermia (n = 153); group 2, low-level leukocytospermia (n = 36), and group 3, frank leukocytospermia, greater than 1 × 10^6^ WBC/mL (n = 22). Compared to the group 1 (0.0 ± 0.01), the leukocyte count was significantly different in group 2 (0.54 ± 0.22; P < 0.001) and group 3 (3.78 ± 3.08; P < 0.001) and between group 2 and group 3 (P < 0.001). There were no significant differences between the three groups in terms of baseline clinical characteristics (Table 1) and conventional semen parameters (concentration, motility, and morphology) (Table 2).

**Table 1:** **Baseline clinical data in three groups of patients categorized by their seminal leukocyte levels**

		Group 1 none	Group 2 0.1–1 × 10 ^6^ WBC/mL	Group 3 >1 × 10 ^6^ WBC/mL	P
Patients		153	36	22	
Age/years		36.95 ± 6.72	38.92 ± 8.13	39.05 ± 7.41	0.34
BMI kg/m^2^		29.49 ± 5.17	28.33 ± 4.71	28.55 ± 4.65	0.21
Volume of Seminal Ejaculate (mL)		3.08 ± 1.32	2.91 ± 1.69	2.87 ± 1.26	0.33
Abstinence (days)		4.03 ± 1.92	4.71 ± 3.64	3.41 ± 1.18	0.25
Infertility Duration (y)		2.22 ± 1.76	3.03 ± 2.65	2.50 ± 2.39	0.37
Infertility Status	Primary	130	24	15	0.012
	Secondary	22	12	7	
Smoking Status	Yes	37	7	1	0.09
	No	113	27	21	
Marijuana Status	Yes	6	4	1	0.21
	No	147	32	21	
Alcohol Status	Yes	93	18	14	0.5
	No	53	16	8	

Group 1 = no leukocytospermia; Group 2 = low level leukocytospermia, Group 3 = frank leukocytospermia, all numbers are reported as mean ± SD.

**Table 2:** **Describing differences in semen parameters, ROS, and DNA damage between the 3 groups of patients**

	Leukocytospermia (× 10 ^6^ WBC/mL)			
	None	0.1–1 million	>1 million	P
Leukocytes (× 10^6^ wbc/mL semen)	0.0 ± 0.01	0.54 ± 0.22^a^	3.78 ± 3.08^a,b^	P < 0.01
Concentration (× 10^6^/mL)	53.04 ± 56.76	69.04 ± 80.72	39.35 ± 39.98	0.97
Motility (%)	48.37 ± 17.42	47.33 ± 25.74	49.23 ± 19.56	0.8
Normal morphology (%)	3.42 ± 3.12	3.56 ± 3.16	4.14 ± 3.79	0.3
ROS (RLU/ sec)	116.7	944.8	61286.8	<0.001
	(49; 550.3)	(127; 3315.4)^a^	(6905; 234876)^a,b^	
DNA damage (%)	19.89 ± 17.31	26.47 ± 19.64^a^	24.60 ± 17.47	0.038

The results are presented as mean ± SD for all the parameters except ROS which is presented as median (25^th^; 75^th^ percentile).

^a^P <0.05 statistically significant compared to non-leukocytospermic group.

^b^P <0.05 statistically significant compared to low-level leukocytospermic group.

### ROS levels

**Figure 2:**
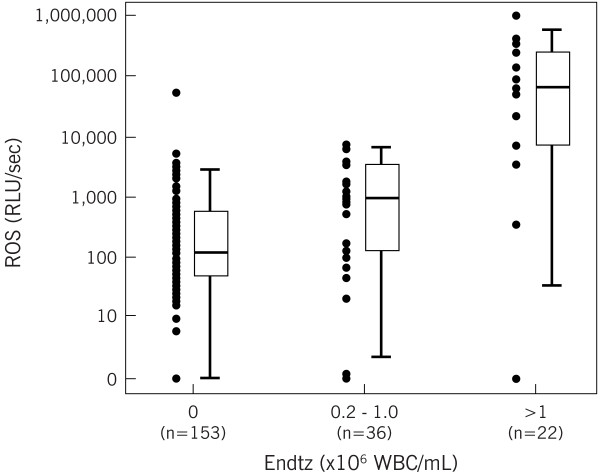
**Box plot demonstrating minimum, maximum, median, and upper and lower quartiles of ROS data.** This box-plot demonstrates the differences in ROS median, range, and upper and lower quartiles. It demonstrates that the median of the leukocytospermic group (median: 1,286.8 RLU/sec) is much higher than both the low leukocytospermic group, and the non-leukocytospermic group. Additionally, the median of the low leukocytospermic group (median: 944.8 RLU/sec) is also much higher than the non-leukocytospermic group (median: 116.7 RLU/sec).

Compared to 63.2% of patients with ROS levels above our reference value (>93 RLU/s/10^6^ sperm), the incidence increased to 81.6% in group 2 (P < 0.016) and 92.3% in group 3 (P < 0.011) and it was comparable between group 2 and group 3. Patients with low-level leukocytospermia (group 2) had significantly higher ROS levels (p = 0.001) and DNA damage (p < 0.05) than patients with no leukocytospermia (group 1) (Table 2). Group 3 (frank leukocytospermia) also had significantly higher ROS levels than group 2 and group 1. Figure 2 demonstrates the differences in ROS levels between the three patient groups.

### DNA fragmentation

**Figure 3:**
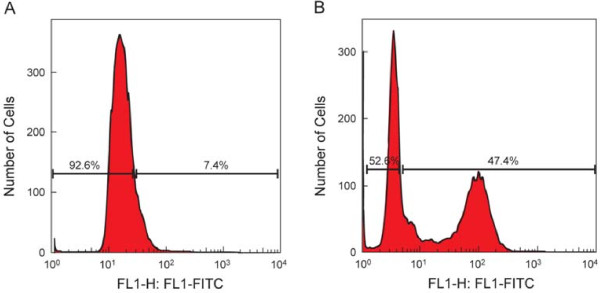
**Flow cytometry results for DNA fragmentation.** DNA damage was measured by flow cytometry, FITC-deoxyuridine triphosphate (dUTP) substrate was added to the TdT enzyme binding with the free 3′-OH termini of the single – and double strand DNA. DNA damage was measured by gating the population of cells as negative or positive. PI stains total DNA and FITC-dUTP stains apoptotic cells. **A**: Representative histogram for a sample that is negative for DNA damage and **B**: Representative histogram for a sample tested positive for DNA damage by flow cytometry.

DNA damage was measured by flow cytometry, using (FITC-dUTP). These results are shown in Table 2. At a reference value of 19% DNA damage, the incidence of DNA damage increased from 39.2% in group 1 to 50% both in group 2 and group 3. However this was not statistically significant. Higher levels of DNA damage was seen in the 0.1-1 × 10^6^ WBC/mL group. Deoxyuridine triphosphate (dUTP) is the substrate that is added by the TdT enzyme to the free 3′-OH break-ends of DNA. DNA damage is measured by gating the population of cells as negative or positive. This is indicated by the percentage distribution of cells that are negative or positive for DNA damage. Figure 3 is a representative example included to illustrate to illustrate the percentage DNA damage in a negative and a positive sample.

### Discussion

In this study only the laboratory parameters were examined and we did not categorize the patients based on their clinical diagnosis. Reactive oxygen species are produced both by morphologically abnormal spermatozoa as well as by white blood cells - especially the granulocytes. Round cells are examined on every sample as part of the semen analysis. According to the WHO guidelines (both 1999 and 2010), round cells are tested for leukocytospermia only if the round cell number on a wet smear is higher than 5 round cells in high power field or greater than 1 × 10^6^. We examined the effects of low-level leukocytospermia (0.1-1.0 × 10^6^ WBC/mL) on sperm concentration, motility and morphology as well as on oxidative stress markers of sperm (ROS levels and DNA damage). We found that while there were no significant changes in semen parameters between the 3 study groups, ROS levels and DNA damage were significantly higher in the low-level leukocytospermia group as compared to the non-leukocytospermia group. This suggests that levels of leukocytospermia lower than the current WHO threshold may impact fertility and may require treatment. It is established that the leukocytes especially the granulocytes are capable of producing significantly higher levels of ROS (>1000 fold) compared to morphologically abnormal spermatozoa with excessive residual cytoplasm in the mid piece [25, 26]. Our study supports the earlier reports that show that low levels of leukocytospermia could also generate ROS [16, 17, 27, 28] and it may have pathological effects [14, 18, 19].

The age in the 3 groups was similar as shown in Table 1. ROS is secreted predominantly by leukocytes, particularly the granulocytes [26]. Morphologically abnormal spermatozoa with mid-piece defects such as presence of extracellular residual cytoplasm also produce ROS. As shown in Table 2, the highest levels of ROS were seen in the leukocytospermic group. In this study we reported only Kruger’s strict morphology criteria and not WHO criteria which include head, midpiece defects and tail abnormalities. Certainly prophylactic measures such as antibiotics may be prescribed to these patients even in cases of moderate leukocytospermia. The patient can be asked to provide 1) another semen sample, 2) undergo a semen culture test and reevaluating a second semen sample is important to see if infection has resolved and leukocyte results are negative.

The WHO establishes the threshold of 1 × 10^6^ WBC/mL as a pathological level to consider treatment of leukocytospermia. Hamada et al. showed that a 3 week course of doxycycline treatment (100 g orally, twice daily) of infertile men with leukocyte levels of 0.2-1 × 10^6^ WBC/mL resulted in significant increases in fertilization rates of patient groups, with pregnancy rates of almost 50% in leukocytospermic samples [29]. Treating infertile men with leukocytospermia has had variable results. Treatment with antioxidants such as a combination of vitamin E, vitamin C, beta-glucan, papaya, and lactoferrin resulted in a decrease in sperm DNA fragmentation [30]. Diet rich in antioxidants β-carotene (5000 IU), vitamin C (60 mg), vitamin E (30 UI), and zinc (15 mg) for at least 3 months as well as fruits and vegetables rich in β-carotene resulted in a significant decrease in ROS level and improvement in sperm DNA integrity in couples with previously reported high ROS and DNA damage. This also resulted in higher pregnancies and fewer miscarriage or abortion rates [31]. Lackner et al. showed that Cox-2 inhibitor therapy improves the sperm concentration and may reduce leukocyte counts in men with abacterial leukocytospermia [32]. Additionally, Yadav et al. showed that treatment with a combination of antibiotic and antioxidant treatment reduced the leukocyte count, significantly improved results of the hypo-osmotic swelling test, and significantly increased the total antioxidant capacity [33]. In that study, there was also significant reduction of nitric oxide (NO) in the seminal plasma.

Henkel et al. examined sperm with and without DNA damage in relation to their success with IVF as well as ICSI [12]. Their results showed a significant correlation between peroxidase-positive cells and ROS, between ROS production and DNA damage, and a tendency towards lower pregnancy rates in women fertilized by DNA-damaged sperm [12]. The effects of leukocytospermia on fertility are still controversial. However, a previous study has shown that high levels of leukocytes in semen resulted in early pregnancy loss with IVF and ICSI as well as in ectopic pregnancies [13]. Both of these are pathologies that are serious to the obstetric patient population, resulting in physical and psychological damage. They were both linked more likely to a male factor, rather than a female factor [13]. While studies specifically linking leukocytospermia to pregnancy are limited, the few available show a relationship. Ignoring the possibility that low-level leukocytospermia has a pathologic effect on male fertility may neglect infertile men with this condition. In fact, low-level leukocytospermia may require testing of semen cultures for possible infection and subsequent treatment to ensure future fertility in some infertile couples.

Leukocytospermia has many implications that may not yet be entirely clear. It is shown to have a relationship with oxidative stress and sperm damage, and an eventual effect on male fertility. Lowering the accepted pathologic level of leukocytospermia may pave the way for infertile men who do not meet WHO standards to receive treatment and may give them a chance to bear biological children. As mentioned, there are already multiple avenues for treatment. These men simply need a cutoff that is more accurate and up to date. ROS are produced mainly by granulocytes [20]. In this study we demonstrated that all samples that were positive for white blood cells, as confirmed by the peroxidase or the Endtz test, were also positive for ROS. Interestingly, we did not see a significant difference in DNA damage in samples from group 2 compared to group 3 even though the levels of ROS were significantly higher in group 3. The plausible reasons for a lack of significant difference between the two groups are: 1) in addition to oxidative stress being a major contributor of DNA damage, apoptosis also contributes to DNA damage. We did not measure apoptosis and also did not examine the clinical diagnosis in these patients. They were categorized solely on the presence or absence of leukocytes. The lack of a significant increase in DNA damage in group 3 compared to group 2 may be attributed to the patient distribution in these two groups; these patients may have adequate antioxidant reserves.

We did not measure the total antioxidant capacity (TAC) in this study. DNA damage caused by apoptosis may not translate into DNA damage as measured by Tunel assay [34]. This is because 1) unlike the somatic cell, spermatozoa nucleus is separated by the neck and the mid-piece, so apoptosis markers cannot reach the nuclear compartment [35]; 2) spermatozoa possess highly truncated base excision repair pathways which are not recognized by the 3-hydroxyl group targeted by terminal transferases in Tunel reaction and 3) nucleus is highly compacted and mitochondria activated endonucleases cannot enter the nuclear compartment. Furthermore spermatozoa do not possess apurinic/apyrimidinic (AP) endonuclease 1 (APE1) to create 3-OH breaks. Therefore Tunel signals are not generated and therefore the damage does not translate into DNA damage.

There was a 100% correlation between Endtz positive sample and ROS production. Earlier we reported ROS-TAC score which is a much stronger predictor of oxidative stress compared to ROS and TAC alone [17, 27]. Leukocytospermic contamination is also associated with a reduced ROS-TAC score. It is difficult to establish a safe minimum WBC count as even low concentration can generate ROS and cause DNA damage [16, 17, 28, 34, 36]. We concluded that the presence of any WBCs is associated with oxidative stress and that these may therefore impair fertility. Complete removal of WBCs from semen samples used for assisted reproduction may help reduce oxidative stress. We had suggested that the WHO defined cutoff of 1 × 10^6^ WBC/mL for leukocytospermia may be too high [16]. As shown in Table 2, both ROS levels and DNA damage was significant in the low level leukocytospermic group when compared to the non-leukocytospermic group.

The WHO cutoff has been challenged previously [14–18]. We are challenging it again, advocating that even men with lower levels of leukocytospermia may have risk factors for infertility, and may require treatment. Based on our present study as well as previous ones, we suggest a new lower threshold for leukocytospermia at 0.1-1.0 × 10^6^ WBC/mL.

This area of study is relatively new--whereas other studies have included low or moderate levels of leukocytospermia in their studies, this is the first study to our knowledge examining the effects of low-level leukocytospermia on oxidative stress markers. We did not examine the association of leukocyte concentration with infection. Studies examining the incidence of bacterial infection with leukocyte concentration are important in further discriminating the presence of low levels of leukoctyospermia and infection. Prescribing antibiotic regimen early on may be helpful in reducing the harmful effects of possible or mild infection. Future studies may look at specific treatment of low level leukocytospermia with antioxidants, anti-inflammatory agents, and possibly antibiotics as well as time-to-pregnancy in couples with low-level leukocytospermia.

The strengths of our study include a sizeable study population. However, our research did not show any significance between non-leukocytospermia patients and those with low level leukocytospermia in terms of DNA damage and we have explained the reason for this lack of significant increase. We expected to see a relationship here, and it was not evident. Further research may need to be undertaken to understand why differences in ROS were significant, but differences in DNA damage were not.

## Conclusions

In conclusion, ROS levels and DNA damage were significantly increased in patients with low-level leukocytospermia. This tells us that there is an important implication to even a reduced number of leukocytes in the semen. We believe that low-level leukocytospermia is pathological and that treatment should be considered in these individuals in addition to semen cultures for underlying infection. The WHO guidelines for leukocytospermia may be too high and may require adjustment.

## Abbreviations

BMI: Body mass index
FITC: Fluorescein isothiocynate
HPF: High power field
ROS: Reactive oxygen species
WBC: White blood cells
WHO: World Health Organization.
